# Intravenous delivery of granulocyte-macrophage colony stimulating factor impairs survival in lipopolysaccharide-induced sepsis

**DOI:** 10.1371/journal.pone.0218602

**Published:** 2019-06-20

**Authors:** Jörg Krebs, Alexander Hillenbrand, Charalambos Tsagogiorgas, Christian Patry, Burkhard Tönshoff, Benito Yard, Grietje Beck, Neysan Rafat

**Affiliations:** 1 Department of Anaesthesiology and Critical Care Medicine, University Hospital Mannheim, University of Heidelberg, Germany; 2 Department of Pediatrics I, University Children’s Hospital Heidelberg, University of Heidelberg, Germany; 3 Department of Medicine V, University Hospital Mannheim, University of Heidelberg, Germany; 4 Department of Anaesthesiology, Helios Dr. Horst-Schmidt Clinic, Wiesbaden, Germany; 5 Department of Neonatology, University Children’s Hospital Mannheim, University of Heidelberg, Germany; 6 Department of Pharmaceutical Sciences, Bahá'í Institute of Higher Education (BIHE), Teheran, Iran; National Institutes of Health, UNITED STATES

## Abstract

**Background:**

Cell-based therapies with bone marrow-derived progenitor cells (BMDPC) lead to an improved clinical outcome in animal sepsis models. In the present study we evaluated the ability of granulocyte macrophage-colony stimulating factor (GM-CSF) to mobilize BMDPC in a lipopolysaccharide (LPS)-induced sepsis model and thereby its potential as a novel treatment strategy.

**Methods:**

Male Wistar rats received LPS (25μg/kg/h for 4 days) intravenously and were subsequently treated with GM-CSF 12.5μg/kg (0h,24h,48h,72h). As control groups, rats were infused with sodium chloride or GM-CSF only. Clinical and laboratory parameters, proinflammatory plasma cytokines as well as BMDPC counts were analyzed. Cytokine release by isolated peripheral blood mononuclear cells from rat spleen upon incubation with LPS, GM-CSF and a combination of both were investigated *in vitro*.

**Results:**

*In vivo*, rats receiving both LPS and GM-CSF, showed a reduced weight loss and increased mobilization of BMDPC. At the same time, this regime resulted in an increased release of proinflammatory cytokines (IL-6, IL-8) and a significantly increased mortality. *In vitro*, the combination of LPS and GM-CSF showed a significantly increased IL-6 release upon incubation compared to incubation with LPS or GM-CSF alone.

**Conclusions:**

GM-CSF did not have a beneficial effect on the clinical course in our LPS-induced sepsis model. It synergistically promoted inflammation with LPS and probably thereby impaired survival.

## Introduction

Septic shock with multiple organ failure is the leading cause of death in intensive care units and remains a major health problem. Despite extensive research and well-conducted clinical trials, no specific novel treatment strategy exists so far [1,2]. Since altered endothelial function and disruption of the vascular barrier is a critical step in the development of multiple organ failure [3–5], endothelial regeneration might have a beneficial effect on the course of sepsis. A potential treatment strategy could be the use of endothelial progenitor cells (EPC) [6,7], which have been shown to play a role [8–12]. An increased concentration of EPC was found in patients with sepsis and acute lung injury, which seem to be involved in endothelial and pulmonary regeneration [8,12] and correlated inversely to disease severity and mortality [8,12].

While initial research indicated that EPC are differentiated into mature endothelial cells and incorporated into the vessel wall to replace the injured endothelium, current understanding proposes different subpopulations of EPC with distinct functions [9]. The endothelial colony forming cells (ECFC) display the ability to form vessels and become part of the systemic circulation of the host animal [13]. The population of bone marrow-derived progenitor cells (BMDPC, e.g. CD34^+^/AC133^+^/KDR^+^), recently also referred to as proangiogenic hematopoietic cells (PHC) [14], are not incorporated into the vessel wall and lack vasculogenic features, but rather exhibit potent paracrine capacity regulating neovascularization via angiogenesis [15]. The mobilization and recruitment of EPC/BMDPC is mediated by soluble factors such as vascular endothelial growth factor (VEGF), granulocyte macrophage colony-stimulating factor (GM-CSF), erythropoietin (EPO) and angiopoetin- (ANG)-2 [16] and offers a particular therapeutic potential. There are two strategies to manipulate the microenvironment to enhance endothelial repair by increasing the number of progenitor cells in the peripheral blood of patients: 1) exogenous administration of BMDPC in form of an allogenic stem cell transplantation, 2) endogenous stimulation of BMDPC release by mobilizing factors, which minimizes immunological complications usually associated with allogenic stem cell transplantation.

In the present study we investigated the ability of GMCSF to mobilize BMDPC in a lipopolysaccharide (LPS)-induced sepsis model and thereby it’s beneficial effect on the clinical course of sepsis.

## Materials and methods

### Animal experiments

This study was approved by the Institutional Review Board for the care of animal subjects (University of Heidelberg, Mannheim, Germany & the Regional Council of Karlsruhe, Germany). All animals received humane care in compliance with the “Principles of Laboratory Animal Care” formulated by the National Society for Medical Research and the stipulations of the German Animal Protection Law in its current version. Twenty-four specific pathogen free male Wistar rats housed in standard conditions with food and water ad libitum were anaesthetized by intraperitoneal (IP) injection of ketamine hydrochloride (100 mg/kg) and xylazine (4 mg/kg). Anaesthesia was maintained with intravenous ketamine via an infusion pump (Braun Perfusor Secura ft, B. Braun Melsungen AG, Melsungen, Germany) at an initial rate of 20 mg/kg/h. The level of anaesthesia was assessed by pinching the paw and tail throughout the experiments. The femoral artery and vein were cannulated with a polyethylene catheter (PE-50, neoLab Heidelberg, Germany) for multiple arterial blood collection and continous intravenous infusion of LPS. Both catheters were subcutaneously tunneled, diverted in the neck and flushed with heparine. After 6 days of convalescence the animals were randomized in 4 groups (Sham, GMCSF, LPS and GMCSF+LPS, n = 6/group) and osmotic minipumps (alzet, OSMOTIC PUMP Model 2ML1, ALZET, Osmotic Pumps, Cupertino, Kalifornien, USA) were implanted subcutaneously and connected to the previously implanted venous catheter.

In the LPS and LPS+GMCSF group pumps were loaded with 0.025mg/kg bodyweight/h LPS (LPS, Escherichia coli O55:B5, Sigma-Aldrich Corporation, Saint Louis, Missouri, USA) and 3 U/ml heparine. The venous catheter was flushed using an equally concentrated fluid bolus of LPS/heparine. In the Sham and GMCSF group the pumps were loaded with isotonic saline and 3 U/ml heparine. The venous catheter was flushed using an equally concentrated fluid bolus of saline/heparine. Additionally the animals in the GMCSF and GMCSF+LPS group received subcutaneous injections of 12.5μg/kg bodyweight GM-CSF (PeproTech GmbH, Zytokine für Deutschland, Hamburg) at 0h, 24h, 48h and 72h after pump implantation. The animals were weighed at 0h and 96h after pump implantation. Arterial blood samples were collected at the same time points for blood gas analysis (Cobas b121, Roche Diagnostics GmbH, Wien, Austria), measurement of the serum chemistry panel [including aspartate aminotransferase (ASAT), alanine aminotransferase (ALAT), bilirubine, lipase, creatinine, urea, creatine kinase using the Hitachi 917 (Roche Diagnostics, Wien, Austria)], proinflammatory cytokines and flowcytometry.

### Enzyme-linked immune-sorbent assay (ELISA)

The concentrations of interleukin (IL)-6, IL-8 and vascular endothelial growth factor (VEGF) were assessed in serum using enzyme-linked immunosorbent assay kits (R&D Systems, Wiesbaden-Nordenstadt, Germany) in triplicate samples. Enzyme-linked immunosorbent assays were performed according to the manufacturer’s instructions.

### Flow cytometry

Peripheral blood mononuclear cells (PBMC) were prepared by gradient centrifugation using Ficoll-Hypaque (Amersham Biosciences, Freiburg, Germany). The expression of cell-surface antigens was determined by two-color immunofluorescence staining using antibodies against CD34 –a marker protein for hematopoietic stem cells- and CD133 –a marker for progenitor cells. CD34^+^/CD133^+^-cells have been described and identified as BMDPC in the literature. In brief, one hundred microliters of PBMC (containing 1 x 10^6^ cells) were incubated at 4°C for 30 mins with 10 μL of goat CD34 polyclonal antibodies (Santa Cruz Biotechnology, Heidelberg, Germany) and 10 μL of rabbit CD133 polyclonal antibodies (Santa Cruz Biotechnology, Heidelberg, Germany). The cells were washed three times to remove unbound antibodies and incubated at 4°C for 30 mins with the secondary antibodies swine anti-rabbit IgG- Fluorescein isothiocyanate isomer 1 (FITC) (DakoCytomation, Hamburg, Germany) and donkey anti-goat IgG (H+L)-Phycoerythrin (PE) (R&D Systems, Wiesbaden-Nordenstadt, Germany). The cells were again washed three times to remove unbound antibodies and finally resuspended in 500 μL of BD FACS lysing solution (BD Biosciences, Heidelberg, Germany). Flowcytometry analysis was performed on a FACSCalibur flow cytometer (BD Biosciences), and the data were analyzed using WinMDI 2•8 software (Scripps Research Institute, La Jolla, CA). A minimum of 300,000 events were collected. Flowcytometry analysis of each probe was performed in triplicate. The frequency of BMDPC in peripheral blood was determined by a two-dimensional side-scatter/fluorescence dot-plot analysis of the samples after appropriate gating. BMDPC counts are expressed as percentage of total PBMC in each rat.

### In vitro cytokine release assay

The PBMC isolated from rat spleen were prepared by gradient centrifugation using Ficoll-Hypaque (Amersham Biosciences, Freiburg, Germany). Five specific pathogen free male Wistar rats weighting 450-500g served as organ donors. The rat spleen was first reduced to small pieces and then homogenized using a mesh (test sieve ∅100mm, Retsch GmbH, Haan, Germany). The obtained spleen cells were then diluted with phosphate buffer saline 1:4 and pipetted onto the Ficoll solution. Centrifugation was performed at 20°C with 1500 rpm for 30 minutes. After that, the interphase was extracted and purified by 3 successive centrifugation steps (each at 20°C with 1300 rpm for 15, 10 and 10 minutes). The PBMC were then seeded in 24-well plates in growth medium (Promocell, Heidelberg, Germany) and stimulated with GM-CSF (PeproTech GmbH, Zytokine für Deutschland, Hamburg) in different concentrations (20ng/ml, 125 ng/ml and 1.25μg/ml), LPS (0.25μg/ml) (Sigma, Deisenhofen, Germany) and the combination of both. After 24 hours, IL-6 and cytokine-induced neutrophil chemoattractant-1 (CINC-1, also IL-8) were measured in the supernatant using ELISA kits (R&D Systems, Wiesbaden-Nordenstadt, Germany) in triplicate samples. ELISA was performed according to the manufacturer’s instructions.

### Statistical methods

All data are presented as mean ± SEM. The statistical comparisons were carried out by means of one-way ANOVA followed by Holm-Sidak’s post-hoc test as required. The survival rates determined according to Kaplan-Meier were compared with the log rank test. The corrections for multiple comparisons were ensured by the Holm-Sidak test. We considered p<0.05 to be statistically significant. Statistical analyses were performed using SigmaPlot 11.0 (Systat Software GmbH, Erkrath, Germany).

## Results

### Weight and blood gas analysis

Weight changes were assessed and blood gas analysis was performed at the beginning of the experiment (0h) and at the end (96h). The results are displayed in Table 1. After 96 hours, the animals in the LPS (p<0.001) and the LPS+GMCSF (p<0.001) group showed a significant weight loss compared to 0h. The weight reduction was more pronounced (p<0.001) in the LPS group compared to the LPS+GMCSF group. Oxygenation expressed as PaO_2_/FiO_2_ ratio (ratio of partial pressure arterial oxygen and fraction of inspired oxygen) was not impaired in any group. On the contrary pH was reduced in the LPS+GMCSF group compared to 0h (p<0.001) and the LPS group (p = 0.006). Ventilation was not compromised, as PaCO_2_ (partial pressure of carbon dioxide) was not altered. Rather we found a decreased base excess in the LPS (p<0.016) and LPS+GMCSF group p<0.001) compared to 0h. Hemoglobin was reduced in all intervention groups (Sham: p<0.006; LPS: p<0.001; GMCSF: p<0.009; LPS+GMCSF: p<0.001) compared to 0h. There was a also significant difference in hemoglobin between LPS and LPS+GMCSF (p = 0.002).

**Table 1:** Weight and blood gas analysis.

		Group			
	base line	Sham	LPS	GMCSF	LPS+GMCSF
	0h	96h	96h	96h	96h
**weight loss (%)**	0	-0.21 ± 0.8	18 ± 1.2 *	-0.59 ± 1.8	6.64 ± 1.7 */**
**PaO**_**2**_**/FiO**_**2**_	444 ± 10	453 ± 4	416 ± 2.5	452 ± 2	421 ± 12
**pH**	7.49 ± 0	7.52 ± 0	7.47 ± 0	7.51 ± 0	7.41 ± 0 */**
**PaCO**_**2**_ **(mmHg)**	37 ± 0.7	34.6 ± 0.4	34.6 ± 0.6	34.7 ± 0.6	33.7 ± 1.2
**BE**	4.1 ± 0.3	4.38 ± 0.5	1.25 ± 0.6 *	4.68 ± 0.8	-1.56 ± 1.2 *
**Hb (g/dl)**	14.7 ± 0.2	11.9 ± 0.1 *	9.35 ± 0.2 *	12.6 ± 0.1 *	11.7 ± 0.5 */**

Sham, 96h after continuous saline infusion; GM-CSF, 96h after continuous saline infusion and daily granulocyte macrophage colony-stimulating factor (GM-CSF) injection; LPS, 96h after continuous lipopolysaccharide infusion; LPS+GM-CSF, 96h after continuous LPS infusion and daily GM-CSF injection

*PaO*_*2*_*/FiO*_*2*_, ratio of partial pressure arterial oxygen and fraction of inspired oxygen; *PaCO*_*2*_, partial pressure of carbon dioxide; *Hb*, hemoglobin; *BE*, base excess; The results are expressed as mean simple linear regression ± SEM

*significant difference vs. base line (0h)

** significant differences vs. GM-CSF *group*.

### Increase of clinically relevant serum parameters

After 96h we found a statistical significant increase for ASAT (p<0.001), creatinine (p<0.001), urea (p<0.001) and creatine kinase (p<0.001) in the LPS+GMCSF group compared to 0h (Table 2). In the LPS group, we found an increase of ASAT (p<0.001), creatinine (p<0.031) and urea (p<0.002).

**Table 2:** Clinically relevant serum parameters.

		Group			
	base line	Sham	LPS	GMCSF	LPS+GMCSF
	0h	96h	96h	96h	96h
**ALAT [U/L]**	55.3 ± 3.8	44 ± 6.9	62 ± 16.3	56.5 ± 9	74.5 ± 19.6
**ASAT [U/L]**	155 ± 24.8	86.2 ± 11.4	446 ± 113 *	135 ± 33.4	492 ± 72.5 *
**Creatinine [mg/dl]**	0.26 ± 0.0	0.24 ± 0.0	0.40 ± 0.1 *	0.27 ± 0.0	0.42 ± 0.1 *
**CK [U/L]**	173 ± 37.2	425 ± 87	447 ± 153	114 ± 21.7	1708 ± 909 *
**Lipase [U/L]**	7.16 ± 0.23	7.24 ± 0.6	6.85 ± 0.6	6.74 ± 0.9	7.14 ± 1.0
**Bilirubine [mg/dl]**	0.07 ± 0.0	0.04 ± 0.0	0.14 ± 0.0	0.00 ± 0.0	0.24 ± 0.2
**Urea [mg/dl]**	44.9 ± 1.4	37.8 ± 1.5	122 ± 18.5 *	46.15 ± 2.1	127 ± 42.4 *

Sham, 96h after continuous saline infusion; GM-CSF, 96h after continuous saline infusion and daily granulocyte macrophage colony-stimulating factor (GM-CSF) injection; LPS, 96h after continuous lipopolysaccharide infusion; LPS+GM-CSF, 96h after continuous LPS infusion and daily GM-CSF injection

*ASAT*, aspartate aminotransferase; *ALAT*, Alanine aminotransferase; The results are expressed as mean simple linear regression ± SEM

* significant difference vs. base line (0h).

### Upregulation of serum concentrations of proinflammatory cytokines

We used ELISA to detect serum concentrations of IL-6, IL-8 and VEGF in the serum of rats in all the groups after 96 hours (Table 3). For IL-6, we found significantly increased concentrations in the LPS (p<0.05) and LPS+GM-CSF (p<0.05) group compared to the base line. In addition, the IL-6 concentration in the LPS+GM-CSF group was significantly increased compared to the concentrations in the LPS group (p<0.05). For IL-8, we found similar results (Table 3). Regarding VEGF, we found significantly increased concentrations in all groups compared to the base line (p<0.05).

**Table 3:** Serum concentrations of proinflammatory cytokines.

		Group			
	base line	Sham	LPS	GMCSF	LPS+GMCSF
	0h	96h	96h	96h	96h
**IL-6 (pg/ml)**	17.2 ± 9.5	28.5 ± 9.2	617 ± 334 *	54.2 ± 21.4	1158 ± 521 */**
**IL-8 (pg/ml)**	21.8 ± 13.8	39.6 ± 11.9	554 ± 149 *	0.0 ± 0.0	900 ± 420 */**
**VEGF (pg/ml)**	5.20 ± 3.3	110 ± 36 *	104 ± 40 *	130 ± 57 *	101 ± 31 *

Sham, 96h after continuous saline infusion; GM-CSF, 96h after continuous saline infusion and daily granulocyte macrophage colony-stimulating factor (GM-CSF) injection; LPS, 96h after continuous lipopolysaccharide infusion; LPS+GM-CSF, 96h after continuous LPS infusion and daily GM-CSF injection

*IL-6*, interleukin 6; *IL-8*, interleukin 8; *VEGF*, *Vascular Endothelial Growth Factor*. The results are expressed as mean simple linear regression ± SEM

*significant differences vs. base line (0h)

**significant differences vs. *LPS group*.

### Upregulation of bone marrow-derived progenitor cells

**Fig 1:**
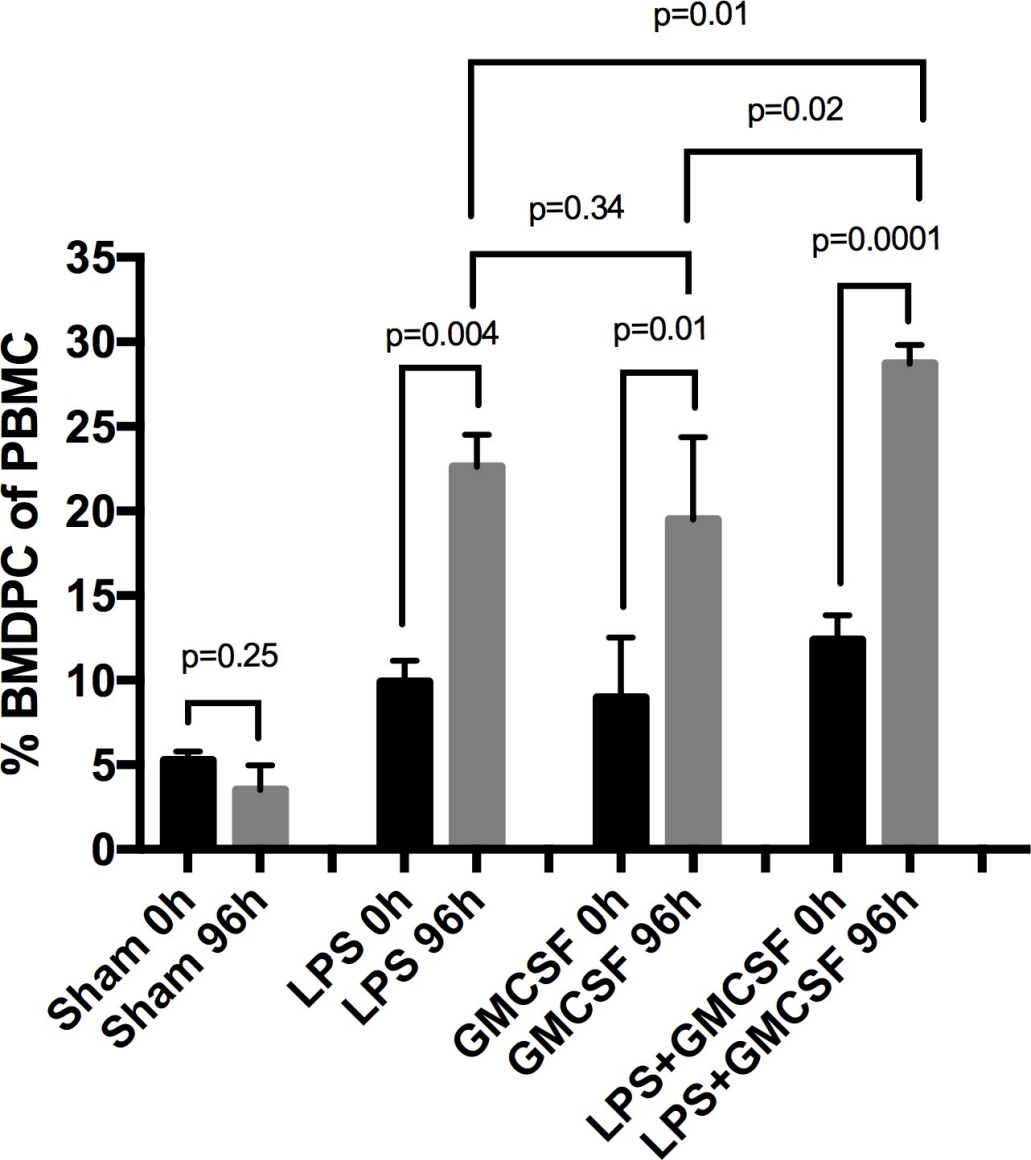
Upregulation of bone marrow-derived progenitor cells (BMDPC). Flowcytometry analysis of the expression of CD34^+^/CD133^+^ cells in the peripheral blood mononuclear cell (PBMC) fraction of rats treated with saline (Sham) (n = 2), lipopolysaccharide (LPS) (n = 3), granulocyte-macrophage colony-stimulating-factor (GM-CSF) (n = 3) or LPS + GM-CSF (n = 6) at two time points (0 and 96 hrs). Significant differences were found between the four groups. The results are expressed as mean simple linear regression ± SEM; p<0.05 was considered to be statistically significant.

We used flowcytometry to detect CD34^+^/CD133^+^-BMDPC in the peripheral blood of rats in the sham (n = 2), LPS (n = 3), GM-CSF (n = 3) and LPS+GMCSF group at two time points (0 and 96 h). Our results showed significant differences in the groups (Fig 1). While there was no significant difference in BMDPC counts after 96 hours in the sham group (5.25±0.54 vs. 3.5±1.46, p<0.25), BMDPC counts in the LPS (9.91±1.25 vs. 22.6±1.91, p<0.004), GM-CSF (8.96±3.56 vs. 19.5±4.86, p<0.013) and LPS+GM-CSF (12.4±1.46 vs. 28.7±1.12, p<0.0001) group were significantly upregulated after 96 hours (Fig 1). In addition, BMDPC counts after 96 hours in the LPS+GM-CSF group were also significantly increased compared to BMDPC counts in the LPS (28.7±1.12 vs. 22.6±1,91, p<0.012) and GM-CSF group (28.7±1.12 vs. 19.5±4.86, p<0.016) after 96 hours (Fig 1).

### Mortality

**Fig 2:**
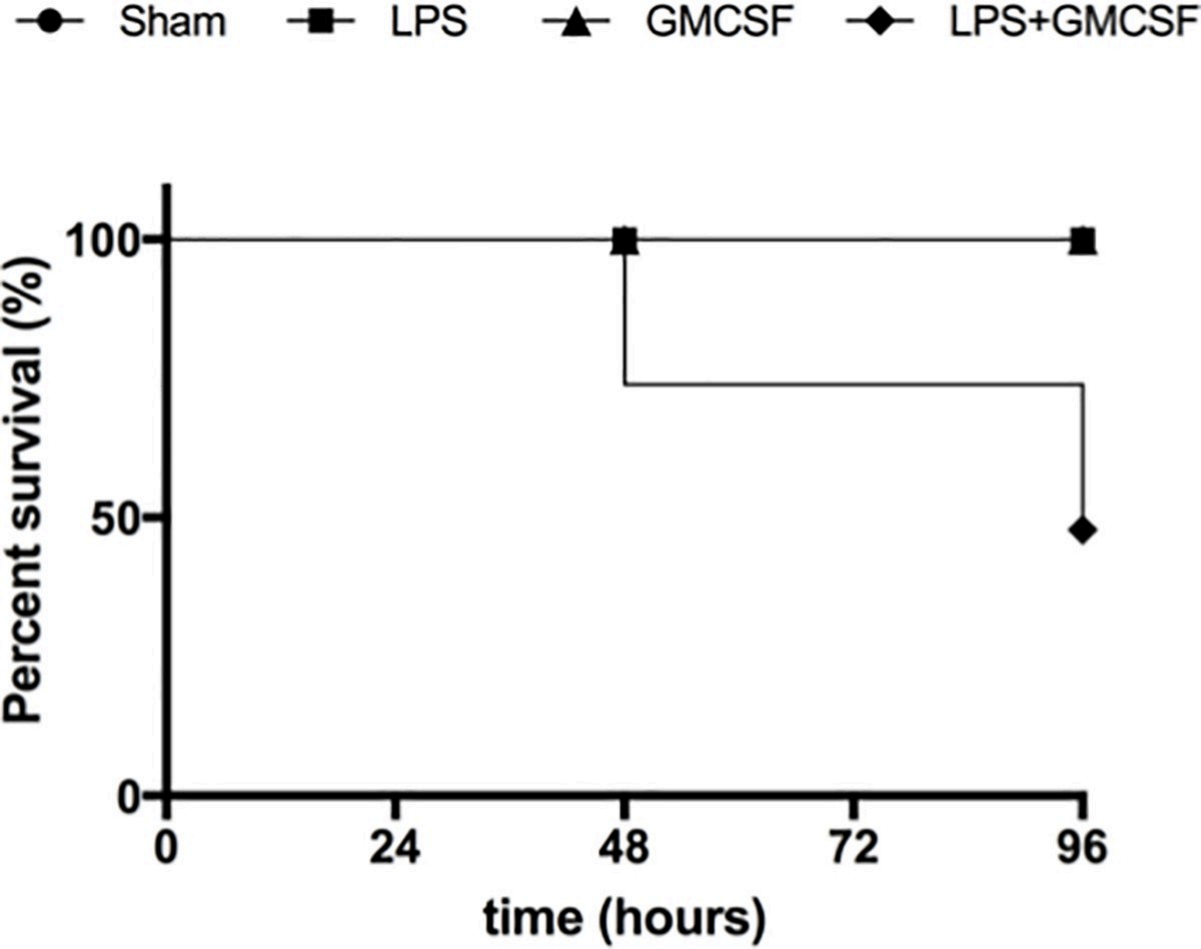
Mortality of the investigated animals. The Kaplan-Meier survival curve compares the mortality of the study groups (LPS+GM-CSF, n = 6; LPS, n, = 6; GM-CSF, n = 6; sham, n = 6). Sham, 96h after continuous saline infusion; GM-CSF, 96h after continuous saline infusion and daily granulocyte macrophage colony-stimulating factor (GM-CSF) injection; LPS, 96h after continuous lipopolysaccharide infusion; LPS+GM-CSF, 96h after continuous LPS infusion and daily GM-CSF injection.

Although aimed as novel treatment strategy, GM-CSF application resulted in a significantly increased mortality in our sepsis-model after 96h. The survival rate of animals in the LPS+GM-CSF group was 33%, while it was 100% in all other groups (p = 0.001) (Fig 2).

### Upregulation of cytokines in vitro

**Fig 3:**
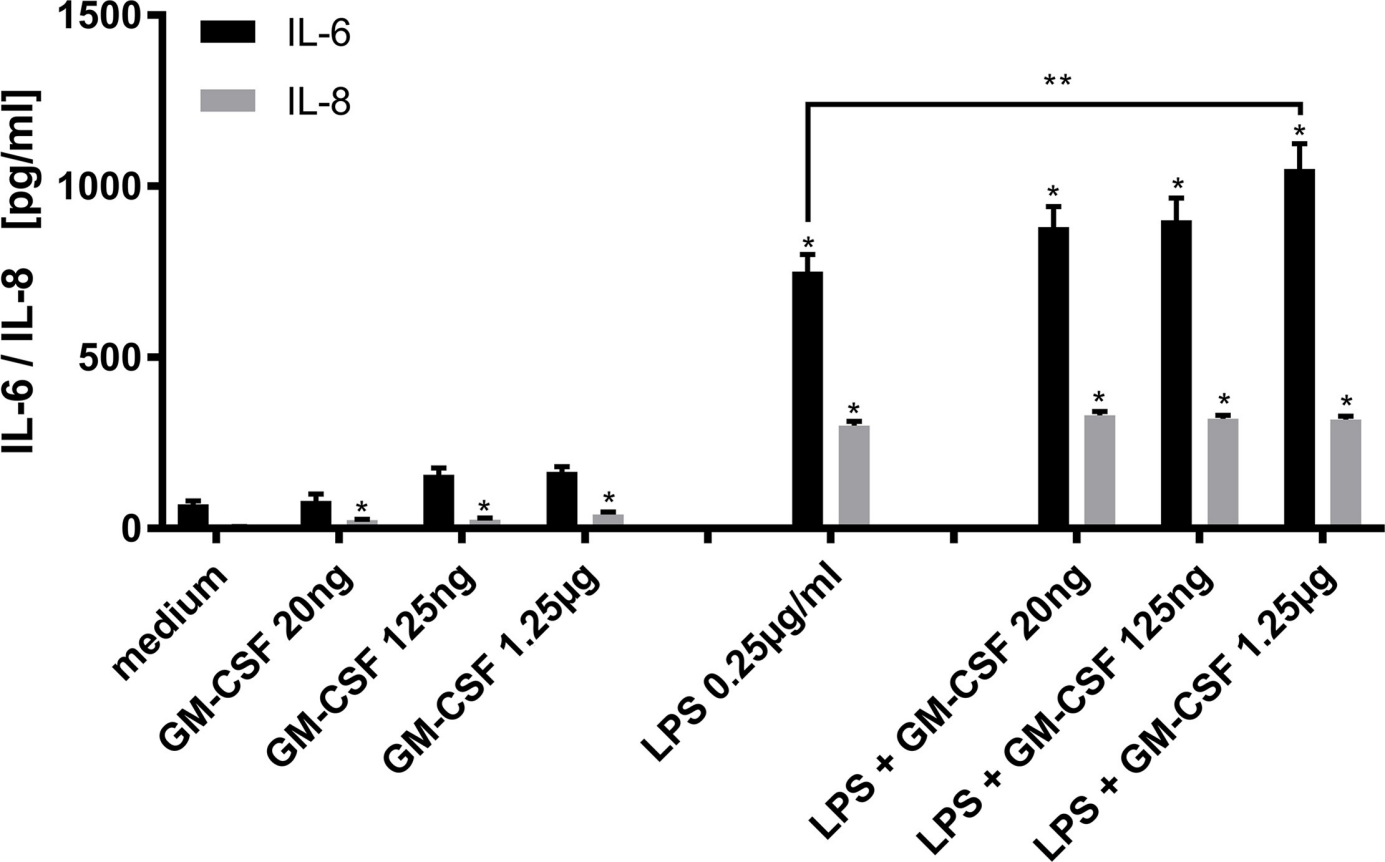
Upregulation of cytokines in vitro. Peripheral blood mononuclear cells (PBMC) were isolated from rat spleen and incubated with medium only, GM-CSF in various concentrations (20ng/ml, 125ng/ml or 1.25μg/ml), LPS (0.25μg/ml) and a combination of LPS (0.25μg/ml) and the different concentrations of GM-CSF. Interleukin-6 (white bars) and Interleukin-8 (grey bars) were measured after 24 hours in the supernatant using ELISA. The results are expressed as mean simple linear regression ± SEM; * p < 0.05 vs. medium, ** p < 0.05 vs. LPS 0.25 μg/ml.

Since GM-CSF application in our *in vivo*-model resulted in increased inflammation markers and mortality, we set out to assess the effect of GM-CSF and LPS alone and in combination on cytokine release *in vitro*. We isolated PBMC from rat spleen and performed ELISA with the supernatant after 24 hour incubation. The addition of GM-CSF in three ascending dosages to the medium resulted in a moderate increase of IL-6 concentration in the supernatant, although this increase did not reach statistical significance in any of the tested GM-CSF concentrations (Fig 3). As expected, the addition of LPS caused a marked increase in IL-6 release (58.9±14.2pg/ml vs. 751±60.3pg/ml, p<0.001) compared to medium (Fig 3). The combination of GM-CSF and LPS increased IL-6 release even further and was statistically significant at the highest dose of GM-CSF compared to LPS incubation alone (751±60.3pg/ml vs. 1029±87.9pg/ml, p<0.05) (Fig 3).

When looking at IL-8, the addition of GM-CSF resulted in a relevant increase of IL-8 for all the GM-CSF concentrations (20ng: 22.2±4.3pg/ml; 125ng: 23±4.0pg/ml; 1,25μg: 47.1±8.0pg/ml), LPS (281±24.2pg/ml) and the three different tested concentrations of LPS+GMCSF (LPS+GMCSF 20ng: 324±20.4pg/ml; LPS+GMCSF 125ng: 321±17.0pg/ml; LPS+GMCSF 1,25μg: 329±20.4pg/ml) compared to the medium (4.34±0.9pg/ml) (all p<0.001). However, there has been no statistical difference between the three different concentrations of LPS+ (Fig 3).

## Discussion

In previous studies, we and others, have demonstrated that progenitor cells are increasingly mobilized from the bone marrow into the circulation in patients with sepsis [8] and acute respiratory distress syndrome (ARDS) [12] and that BMDPC transplantation in animal models of sepsis and ARDS lead to an improved clinical course and a reduced mortality [10,11]. To minimize immunological complications of allogenic BMDPC transplantation, endogenous stimulation of BMDPC release by administration of GM-CSF might be an alternative. Our findings demonstrated that administration of GM-CSF results in an increased mobilization of BMDPC in LPS-induced sepsis, but surprisingly impairs survival significantly. The animals in the LPS+GM-CSF group showed a significant weight loss, mildly increased markers of liver and kidney failure and rhabdomyolysis and an increased release of IL-6 and IL-8. *In vitro*, we demonstrated that incubation of isolated PBMC from rat spleen with LPS and GM-CSF simultaneously results in a significantly increased IL-6 release.

Already 25 years ago, the use of G-CSF or GM-CSF treatment in animal models of bacteraemia was investigated [17,18]. Based on studies showing that recombinant human (rh)G-CSF induce neutrophilia and modulate neutrophil proliferative and neutrophil storage pools in the newborn rat, Cairo et al. investigated the adjuvant effect of rhG-CSF given to group B streptococcus (GBS) septic newborn (less than 36 h) rats treated with and without antibiotic therapy [17]. The animals receiving rhG-CSF and antibiotics had a significantly increased survival rate after 72 hours compared to control animals and animals receiving either rhG-CSF or antibiotics only [17]. Additionally, when rhG-CSF was administered prophylactically (6 h before GBS), a similar significant synergistic effect in survival was demonstrated with G-CSF plus antibiotics versus antibiotics alone [17]. Therefore, it was concluded that either simultaneous or prophylactic pulse administration of rhG-CSF may have a synergistic and protective effect on survival in antibiotic-treated experimental GBS in the neonatal rat.

In contradiction to these results, we have found an increased mortality by GM-CSF application in our sepsis-model. We could demonstrate that animals, which received LPS and GM-CSF, released increasing amounts of pro-inflammatory cytokines and confirmed these results *in vitro* by incubating PBMC from rat spleen with LPS and GM-CSF, which has also lead to an increased release of proinflammatory cytokines. It has been shown in the past, that GM-CSF does induce proinflammatory cytokine release, especially IL-6 and IL-8, in macrophages [19,20]. Although also stimulating the mobilization of BMDPC, GM-CSF seems to intensify the inflammatory response induced by LPS in a synergistic manner. In this short time of clinical impairment, the increased numbers of BMDPC in the circulation cannot exert their beneficial effects and do not come to fruition.

With regard to ARDS, there are conflicting results on the effect of GM-CSF. Goodman et al. have demonstrated that plasma from atients with ARDS enhanced PBMC viability, while blocking the GM-CSF receptor significantly reduced PBMC viability in ARDS plasma, but not in normal plasma [21]. They suggested that GM-CSF receptor blockage might be a novel therapeutic approach for ARDS treatment [21]. Matuete-Bello et al. confirmed that bronchio-alveolar lavage fluid (BALF) from patients on days 1 and 3 of ARDS showed inhibition of neutrophil apoptosis, but BALF from patients at later stages of ARDS, or from patients at risk for ARDS, did not [22]. Consistently, they found increased concentrations of G-CSF and GM-CSF in BALF at early stages and decreased concentrations at later stages of ARDS [22]. However, their observations showed a significant association of higher concentrations of GM-CSF in BALF of ARDS patients with improved survival [22]. In a different study, BAL concentrations of GM-CSF, G-CSF and IL-8 were increased in ARDS patients compared to healthy individuals, but concentrations of GM-CSF were much lower than those of G-CSF and IL-8 [23]. Levels of G-CSF and IL-8, but not GM-CSF, correlated significantly with each other and with BAL neutrophil counts, and only levels of G-CSF were significantly higher in non-survivors than survivors indicating a role in the pathogenesis of ARDS [23].

In our present study, GM-CSF treatment resulted in an increased mortality of animals. Although BMDPC were increasingly mobilized in response to GM-CSF administration, they were not able to prevent the severe pro-inflammatory effects of the GM-CSF treatment. In line with these observations, levels of G-CSF mRNA correlated with severity of shock, infiltration of polymorphonuclear leukocytes (PMN), pulmonary edema and hypoxia in a rat model of hemorrhagic shock [24,25]. The same group instilled G-CSF into the lungs by intratracheal injection to determine whether increased tissue levels of G-CSF contribute to PMN recruitment and PMN-mediated injury [26]. Animals treated with G-CSF became hypoxic, hypocapnic, and alkalotic and demonstrated increased BAL fluid cellularity compared with control animals [26]. Histological examination of the lungs from G-CSF-treated rats revealed marked edema and increased PMN within the interstitium and alveoli [26]. The authors concluded that these results indicate that the presence of G-CSF alone in the lung can lead to recruitment of PMN, lung injury, and impaired pulmonary function, suggesting that local production of G-CSF may contribute to the development of lung damage and possibly ARDS in the setting of resuscitated hemorrhagic shock. In line with the hypothesis that G-CSF/GM-CSF treatment is associated with the development of lung damage, Verhoef et al. reported a case of a patient who developed ARDS during treatment with rhGM-CSF for severe transfusion-dependent refractory anemia with excess of blasts [27].

Conflicting results in regard to the effect of G-CSF/GM-CSF have also been described for sepsis. In pre-clinical models of sepsis in rats, GM-CSF correlated positively with the survival outcome [28]. A meta-analysis investigating the effects of G-CSF and GM-CSF therapy in non-neutropenic patients with sepsis identified twelve randomized control trials (RCT) with 2.380 patients [29]. There was no significant 28-day mortality, in-hospital mortality or adverse events when G-CSF or GM-CSF were compared with placebo [29]. However, G-CSF or GM-CSF therapy significantly increased the reversal rate from infection. Another meta-analysis investigating the effects of G-CSF and GM-CSF therapy in sepsis included four RCTs with 154 patients [30]. In accordance with the previous study, there was no significant difference in 28-day mortality or rate of adverse events as well as no significant difference in length of stay in hospital or intensive care units (ICU) and sepsis-related organ failure assessment score between the GM-CSF treatment and traditional therapy [30]. Therefore, there is no current evidence supporting the routine use of GM-CSF in septic patients. A recent randomized trial in very preterm small-for-gestational age (SGA) babies investigated whether the administration of GM-CSF could prevent neonatal sepsis and produce differences in survival free of severe disability [31]. The authors found no significant differences in health outcomes or health and social care costs between the trial groups at two years of age [31]. Marginally, more children receiving GM-CSF were reported to have cough and had signs of chronic respiratory disease, though this was not reflected in bronchodilator use or need for hospitalisation for respiratory disease [31]. Therefore, they also concluded that the administration of GM-CSF to very preterm SGA babies is not associated with improved or more adverse outcomes [31].

The limitations of this study are the limited number of experiments and the lack of GM-CSF receptor blockade studies. The results from this study remain therefore associative and loosely mechanistic. To interpret the results of our study within a clinical context and with respect to the design of future *in vivo* studies, we also have to discuss the limitations of our experimental set-up. Our endotoxemia model induced by LPS is obviously not identical with an infection caused by a pathogen, in which LPS leads to an activation of the innate immune system to eliminate the pathogen. Also, the survival rate of the animals in the LPS group was hundred percent, which already shows, that the endotoxemia did only cause a mild sepsis. We can only speculate that GM-CSF application could lead to different results in a model of severe sepsis. Another limitation of our set-up relates to the application of GM-CSF simultaneously to the induction of sepsis, which does not reflect the clinical setting, where an application is only possible after sepsis has been manifested. A respective prolonged set-up might have revealed different result. Also, we have not tested a treatment strategy based on G-CSF, which could have also led to a different outcome, since previous studies showed a differing impact by GM-CSF and G-CSF [21]. Another important aspect is, that augmented cytokine production might not be the only reason that might affect survival rate in our model. The available data in our study is unfortunately limited and discussing other influencing factors would only be speculation. Taken together, the interpretation of our results with respect to clinical implications must be made carefully. However, we suggest to test alternative growth factors to enhance endogenous BMDPC mobilization.

## Conclusions

**Fig 4:**
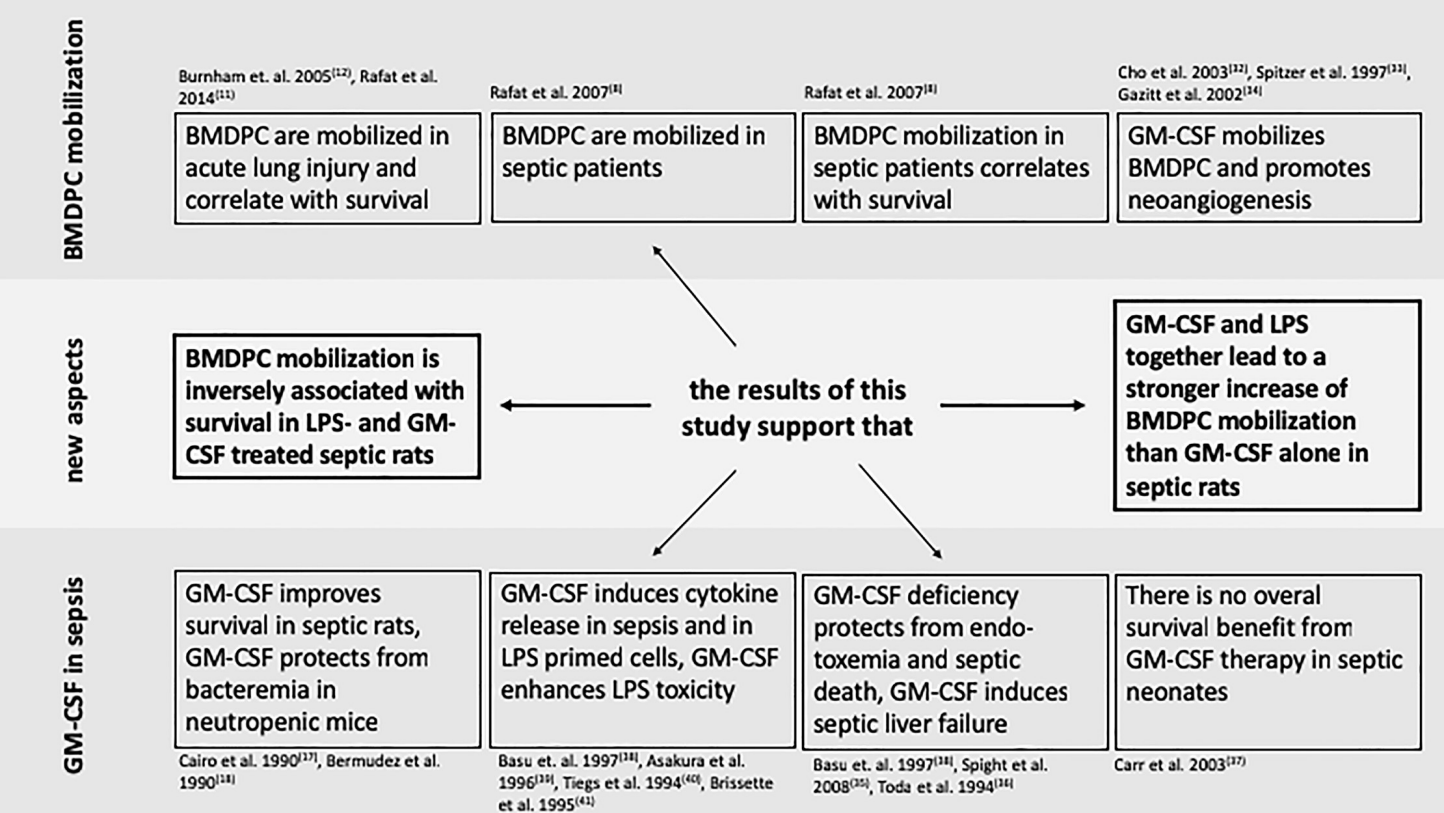
New findings of this study in comparison with previous literature. The new findings of our current study are highlighted and an overview of the findings of previous studies in regard to BMDPC mobilization and GM-CSF application in sepsis is presented. The references to the studies are found in brackets.

We have demonstrated that GM-CSF administration not only results in an increased mobilization of BMDPC, but surprisingly impairs survival significantly in our experimental model. There is indication that GM-CSF administration amplifies the LPS-induced release of proinflammatory cytokines, which could also be confirmed *in vitro*. Fig 4 gives an overview of our findings in comparison to the previous literature [32–41]. Due to our limited results, we would suggest that GM-CSF does not qualify as a novel treatment option to increase endogenous mobilization of BMDPC to improve the clinical course in sepsis. Future studies will have to investigate other mobilizing factors to increase BMDPC numbers endogenously.
